# Improved bladder contractility after transplantation of human mesenchymal stem cells overexpressing hepatocyte growth factor into underactive bladder from bladder outlet obstruction models of rats

**DOI:** 10.1371/journal.pone.0261402

**Published:** 2021-12-22

**Authors:** Jae Heon Kim, Hee Jo Yang, Sung Sik Choi, Seung U. Kim, Hong J. Lee, Yun Seob Song

**Affiliations:** 1 Department of Urology, Soonchunhyang University School of Medicine, Seoul, Republic of Korea; 2 Department of Urology, Soonchunhyang University School of Medicine, Cheonan, Republic of Korea; 3 Medical Science Research Institute, Chungbuk National University, Cheong Ju, Republic of Korea; 4 Division of Neurology, Department of Medicine, UBC Hospital, University of British Columbia, Vancouver, Canada; University College London Institute of Child Health, UNITED KINGDOM

## Abstract

**Introduction:**

An underactive bladder can lead to difficulty in voiding that causes incomplete emptying of the bladder, suggesting the need for a new strategy to increase bladder contractility in such patients. This study was performed to investigate whether human mesenchymal stem cells (hMSCs) were capable of restoring bladder contractility in rats with underactive bladder due to bladder outlet obstruction (BOO) and enhancing their effects by overexpressing hepatocyte growth factor (HGF) in hMSCs.

**Materials and methods:**

The hMSCs were transplanted into the bladder wall of rats. Fifty female Sprague-Dawley rats at six weeks of age were divided into five groups: group 1: control; group 2: sham intervention; group 3: eight-week BOO; group 4: BOO rats transplanted with hMSCs; and group 5: BOO rats transplanted with hMSCs overexpressing HGF. Two weeks after the onset of BOO in groups 4 and 5, hMSCs were injected into the bladder wall. Cystometry evaluation was followed by Masson’s trichrome staining of bladder tissues. Realtime PCR and immunohistochemical staining were performed to determine for hypoxia, apoptosis, and angiogenesis.

**Results:**

Collagen deposition of bladder increased in BOO but decreased after transplantation of hMSCs. The increased inter-contraction interval and residual urine volume after BOO was reversed after hMSCs transplantation. The decreased maximal voiding pressure after BOO was restored by hMSCs treatment. The mRNA expression of bladder collagen1 and TGF-β1 increased in BOO but decreased after hMSCs transplantation. The decrease in vWF-positive cells in the bladder following BOO was increased after hMSCs transplantation. Caspase 3 and TUNEL-positive apoptosis of bladder cells increased in BOO but decreased after transplantation of hMSCs. These effects were enhanced by overexpressing HGF in hMSCs.

**Conclusion:**

Transplantation of hMSCs into bladder wall increased the number of micro-vessels, decreased collagen deposition and apoptosis of detrusor muscle, and improved bladder underactivity. The effects were enhanced by overexpressing HGF in hMSCs. Our findings suggest that the restoration of underactive bladder using hMSCs may be used to rectify micturition disorders in patients following resolution of BOO. Further studies are needed before hMSCs can be used in clinical applications.

## Introduction

Older male individuals may experience symptoms of lower urinary tract obstruction primarily due to benign prostatic hyperplasia. Long-term bladder outlet obstruction (BOO) is initiated via progression from a compensated state with normal elimination to a decompensated state with diminished flow, discontinuous urination, resulting in postvoid residual urine volume. The bladder capacity to eliminate urine is primarily affected by suboptimal activity of the detrusor muscle.

An underactive bladder can lead to difficulty in voiding and cause incomplete emptying of the bladder. In the absence of effective treatments to improve detrusor function and thereby facilitate bladder emptying, the management generally entails bladder drainage or therapies aimed at reducing bladder outlet resistance. Cholinergic agents may enhance bladder contractility. However, it is often difficult to manage voiding disorders using medications alone in patients with underactive bladder. Clean intermittent catheterization can also be used to enhance bladder contractility. However, it can increase the risk of urinary tract infection. In addition, some patients refuse catheterization because of pain. Therefore, a new strategy to increase bladder contractility in patients with underactive bladder is desired.

Impaired detrusor muscle associated with ischemia, apoptosis, and bladder fibrosis have been found in underactive bladder induced by BOO [1–9]. Therefore, treatment of ischemia and inhibition of bladder fibrosis and apoptosis are required.

Mesenchymal stem cells (MSCs) can regenerate and differentiate into a wide range of cell types. MSCs synthesize growth factors and alleviate fibrosis [10]. Evidence indicates that transplantation of MSCs can increase the blood levels of hepatocyte growth factor (HGF) and stimulate remnant hepatic renewal [11]. HGF released from MSCs prevent apoptosis of neurons or muscles [12, 13]. The therapeutic potential of MSCs in experimental cardiac or liver fibrosis has been reported previously [11, 14].

In a recent study, Boga et al. [15] investigated bladder function improvement using MSCs derived from neonate bladder in a rat model of diabetes mellitus (DM) and reported symptomatic improvement in diabetic bladder dysfunction. Liang et al. [16] also reported the therapeutic effect of amniotic fluid stem cells in a rat model of bladder dysfunction in DM model. We previously reported the role of MSCs in a model of bladder dysfunction caused by spinal cord injury and BOO [17, 18]. This study was performed to investigate whether human mesenchymal stem cells (hMSCs) restore bladder contractility in rats with underactive bladder and enhance their effects by overexpressing HGF in hMSCs.

## Materials and methods

### Stable human MSC cell line

BM3.B10, a stable immortalized bone marrow hMSC line, was generated via transfection of human fetal bone marrow stem cells with amphitropic replication-incompetent retroviral vector encoding v-myc. Human fetal bone marrow MSCs were infected with a retroviral vector encoding v-myc oncogene transcribed from mouse leukemia virus long terminal repeat (LTR) alongside gene encoding resistance to neomycin transcribed from a SV40 early promotor without amphitropic replication capacity. The goal was to stimulate the propagation of a stable and immortalized human bone marrow MSCs. Additional studies of hMSCs were performed among the different clones classified as bone marrow 3 (BM3) human MSC cell lines [17, 18].

### Human HGF gene transfection into MSCs

LipofectAMINE (Invitrogen, Carlsbad, CA, USA) was used to transfect PT67 mouse packaging cell line with pLPCX-HGF vector. Puromycin (3-μg/mL) was used to select a stable PT67 cell line for three days. The hMSCs were transfected with a replication-incapable retroviral vector derived from PT67. The HGF gene transfection was followed by isolation, screening, expansion, and transplantation of puromycin-resistant clones [17, 18].

### Experimental model of BOO

Every experimental procedure was approved by the Institutional Animal Care and Use Committee of Soonchunhyang University, Seoul Hospital (IRB no:2019–4) and performed in accordance with the National Institute of Health Guide for the Care and Use of Laboratory Animals. The empirical study involved 50 female Sprague-Dawley rats aged six weeks and weighting 200 g each. They were divided equally into five groups: control (group 1), sham intervention (group 2), BOO (group 3), transplantation of hMSCs at 14 days post-BOO (group 4), and transplantation of hMSCs overexpressing HGF at 14 days post-BOO (group 5). The BOO procedure was performed after isoflurane anesthesia was administered. The skin in the inferior abdomen was incised to facilitate urethral dissection, followed by 4–0 silk sutures surrounding the urethra, including a metal rod measuring 1-mm in external diameter in an extraluminal position. This rod was removed when the suture was tied and the abdominal wall was closed. A 500-μL syringe with a 26G needle was used to inject around 1.0 x 10^6^ hMSCs cells into the bladder wall of rats in group 4 and 1.0 x 10^6^ hMSCs overexpressing HGF cells into the bladder wall of rats in group 5 after 14 days post-BOO. Infection was avoided by administering 10 mg/kg of Flomoxef (cephalosporin; Ildong, Seoul, South Korea) every day.

### Post-transplantation enhancement of voiding function

Voiding response was evaluated at six weeks post-transplantation. Isoflurane was used to anesthetize female Sprague-Dawley rats, followed by a midline abdominal incision to expose the bladder. A small incision was made in the bladder dome to insert a Polyethylene (PE)-50 catheter. The bladder end of this catheter was heated to create a collar for tight suture. The other end of the catheter passed through the subcutaneous tissue and emerged out of the skin. The muscle and skin were sutured to close the abdominal incision. Subsequently, rats intended for investigation without anesthesia were held in a restraining cage for 5–6 hours, including two hours to recover from isoflurane anesthesia. A T-stopcock was used to connect the bladder catheter to a pressure transducer and a pump for continuous administration of physiological saline. To induce repetitive voiding response, physiological saline was administered at a fixed rate of 0.04 mL/min under ambient temperature. Measurements were performed to determine the inter-contraction interval, which is the duration between voids or reflex bladder contractions, the maximal voiding pressure, pressure threshold, and residual urine volume.

### Histology and immunohistochemistry

Upon completion of cystometry, bladder was extracted from five rats in each group. Bladders were weighed after removing surrounding tissues, followed by freezing and storage in liquid nitrogen until biochemical and molecular biological investigations were performed. The remaining five rats in each group were subjected to cardiac perfusion with cold saline (100 ml) and 4% paraformaldehyde (100 ml) in phosphate-buffered saline (PBS). Their bladders were fixed in 4% paraformaldehyde for 24 hours, followed by cryoprotection in 30% sucrose for 24 hours. A cryostat (Leica CM 1900) was used to section bladders into 20-μm segments. Hematoxylin and Eosin (H&E) and Masson’s trichrome were then used for staining. A microscope was used to analyze slides and capture ten representative zones selected arbitrarily from light microscope images. A color monitor facilitated visualization of the captured video images. Digitization and analysis were concomitantly performed using an IBM computer. A square micrometer was used for blinded calculation of representative areas in every slide to assess the outcomes of Masson’s trichrome staining, with the mean area expressed as a relative percentage. The mean percentage of collagen area was determined using the following formula: (collagen) / (collagen + muscle) based on area of smooth muscle and connective tissue stained red and blue, respectively. OPTIMAS version 6.1 (Media-Cybernetic, Bethesda, MD, USA) was used for quantitative image analysis.

Immunofluorescence staining was performed to determine the transplanted hMSCs using human nuclear matrix antigen (hNuMA, 1:100 dilution, Abcam, Cambridge, MA, USA) and neighboring serial sections. Immunostaining was performed using antibodies against von Willebrand Factor (vWF, 1:200 dilution, Ongogene, Cambridge, MA, USA) and caspase-3 (Cas3, 1:200 dilution, Santa Cruz Biotechnology, Dallas, TX, USA). Deoxynucleotidyl transferase-mediated dUTP nick end labeling (TUNEL) and apoptosis detection kit (Trevigen Inc, Gaithersburg, MD, USA) were also used. A ten-minute heating procedure with 9-mM sodium citrate (pH 5.0) was performed to retrieve antigen in the case of vWF, Cas3, and TUNEL. Peroxidase blocking reagent (Dako, Carpinteria, CA, USA) was used to inhibit the activity of endogenous peroxidase. Free-floating sections of bladder segments were incubated overnight with a mixed primary antibody solution at 4°C, followed by incubation with a mixed secondary antibody solution of Alexa Fluor 488- conjugated anti-mouse IgG (1:1000 dilution, Molecular Probe, Eugene, OR, USA) and Alexa Fluor 594-conjugated anti-rabbit IgG (1:1000 dilution, Molecular Probe, Eugene, OR, USA) at ambient temperature for 60 minutes. Negative control sections were obtained similarly from every rat without using primary antibodies. The stained sections were analyzed with an Olympus laser confocal fluorescence microscope.

### mRNA expression

TRIzol® reagent (Invitrogen, Carlsbad, CA, USA) was used to extract the total RNA from cells and samples. Primers specific for collagen1, transforming growth factor- β1 (TGF-β1), HGF, and glyceraldehyde 3-phosphate dehydrogenase (GAPDH) were used to perform real-time PCR (Table 1). Quantification of relative gene expression was based on threshold cycle value. Gene expression was normalized against the expression of β-actin, a housekeeping gene. Data are presented as means of three experiments. Real-time PCR was performed under the following conditions: 10 seconds at 95°C, 40 cycles of five seconds each at 95°C, followed by 30 seconds at 60°C. At the end of real-time PCR, melting curve analysis was carried out at a temperature range of 60–95°C.

**Table 1:** Primers used in real-time PCR.

Genes	Forward	Reverse
**Collagen1**	TACAGCACGCTTGTGGATGG	CAGATTGGGATGGAGGGAGTT
**TGF-β1**	CTCCCGTGGCTTCTAGTGC	GCCTTAGTTTGGACAGGATCTG
**HGF**	GAATGCATGACCTGCAACGG	TGTCGGGATATCTTTCCGGC
**β-actin**	GCACCACACCTTCTACAATG	TGCTTGCTGATCCACATCTG

### Statistical analysis

Two-way ANOVA and post-hoc Tukey test were used to analyze the stem cell transplantation. The values were expressed as mean ± standard error (SE). Statistical significance was considered when p value was less than 0.05.

## Results

### Body and bladder weights

**Fig 1:**
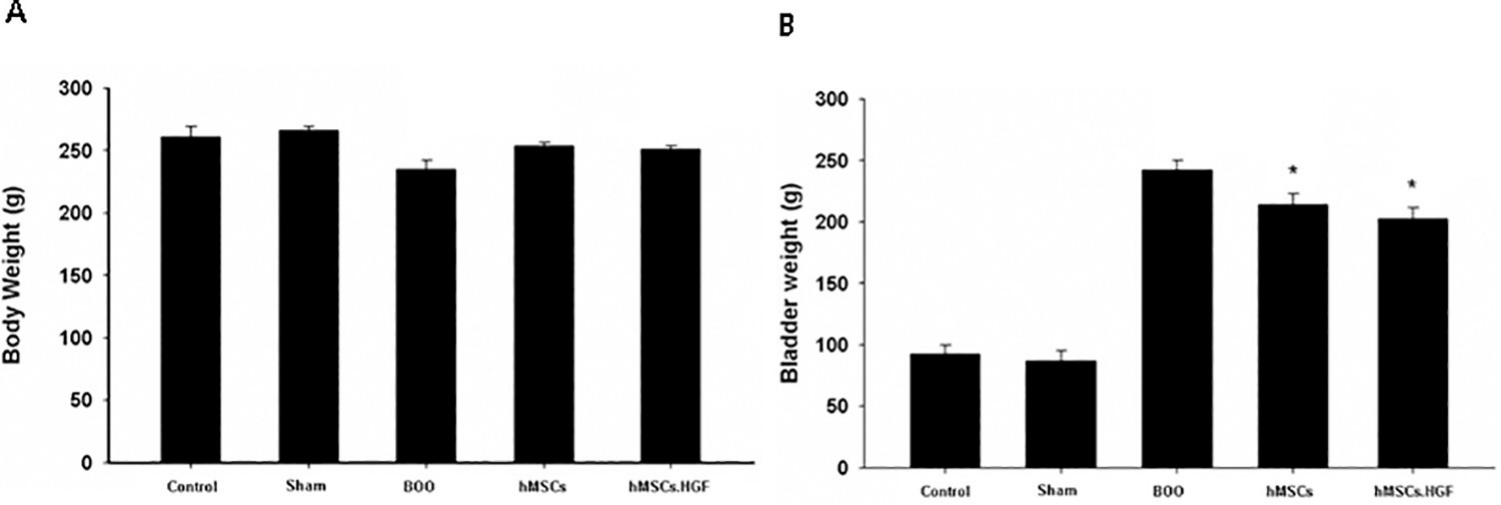
Change in body and bladder weight after transplantation. A. There was no significant difference in body weight between groups. B. The bladder weight was increased in the group with BOO than in the sham operation group. The group transplanted with hMSCs and HGF-overexpressing hMSCs showed decreased bladder weight than the group with BOO. Sham = sham operation, BOO = bladder outlet obstruction, hMSCs = human mesenchymal stem cells, hMSCs.HGF = HGF overexpressing human mesenchymal stem cells. * *p* < 0.05.

The body weights of groups 1,2,3,4, and 5 were 261 ± 8.5 g, 266 ± 3.3 g, 235 ± 7.1 g, 254 ± 2.4 g, and 251 ± 2.7 g, respectively, suggesting no significant difference in body weight between groups. The bladder weights of groups 1, 2, 3, 4, and 5 were 92.8 ± 7.2 mg, 86.9 ± 8.5 mg, 242.2 ± 7.9 mg, 214.2 ± 8.7 mg, and 207.2 ± 7.9 mg, respectively. Compared with sham intervention and control groups, the BOO group showed higher bladder weight. The bladder weight was significantly (*p* < 0.05) lower in the hMSC transplantation group (group 4) than in the BOO group without hMSC transplantation (group 3). Bladder weight was further decreased in the group transplanted with HGF-overexpressing hMSCs (group 5) (*p* < 0.05) (Fig 1).

### Collagen area

**Fig 2:**
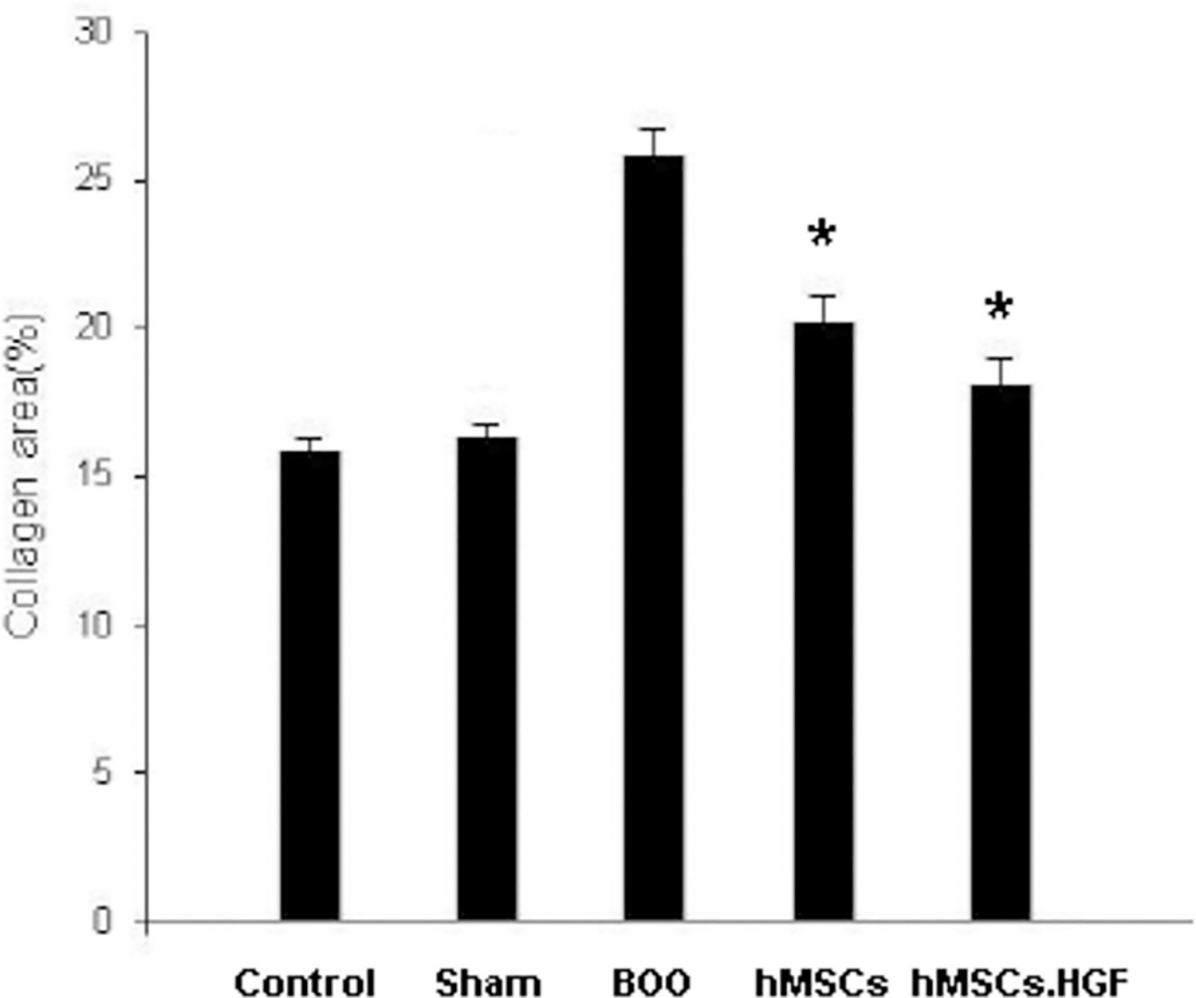
Percentage of collagen area. The group with BOO showed increased ratio of collagen to collagen + muscle (%) than the group with sham operation and control (*p<0.05). The group with BOO transplanted with hMSCs showed decreased ratio than the group with BOO. The effect was enhanced by HGF overexpression of hMSCs. Sham = sham operation, BOO = bladder outlet obstruction, hMSCs = human mesenchymal stem cells injection, hMSCs.HGF = HGF overexpressing human mesenchymal stem cells injection. *p < 0.05.

Comparing sham intervention and control groups, the BOO group had a higher ratio of collagen to collagen + muscle (%) (*p* < 0.05). The BOO group transplanted with hMSCs (group 4) showed a lower ratio of collagen to collagen + muscle (%) than the BOO group (group 3). The ratio of collagen to collagen + muscle (%) was the lowest in the group transplanted with HGF-overexpressing hMSCs (group 5) (*p* < 0.05). Masson’s trichrome staining validated the increased collagen deposition in the BOO group. However, the transplantation of hMSCs reduced collagen deposition (Fig 2).

### Post-transplantation improvement of voiding function

**Fig 3:**
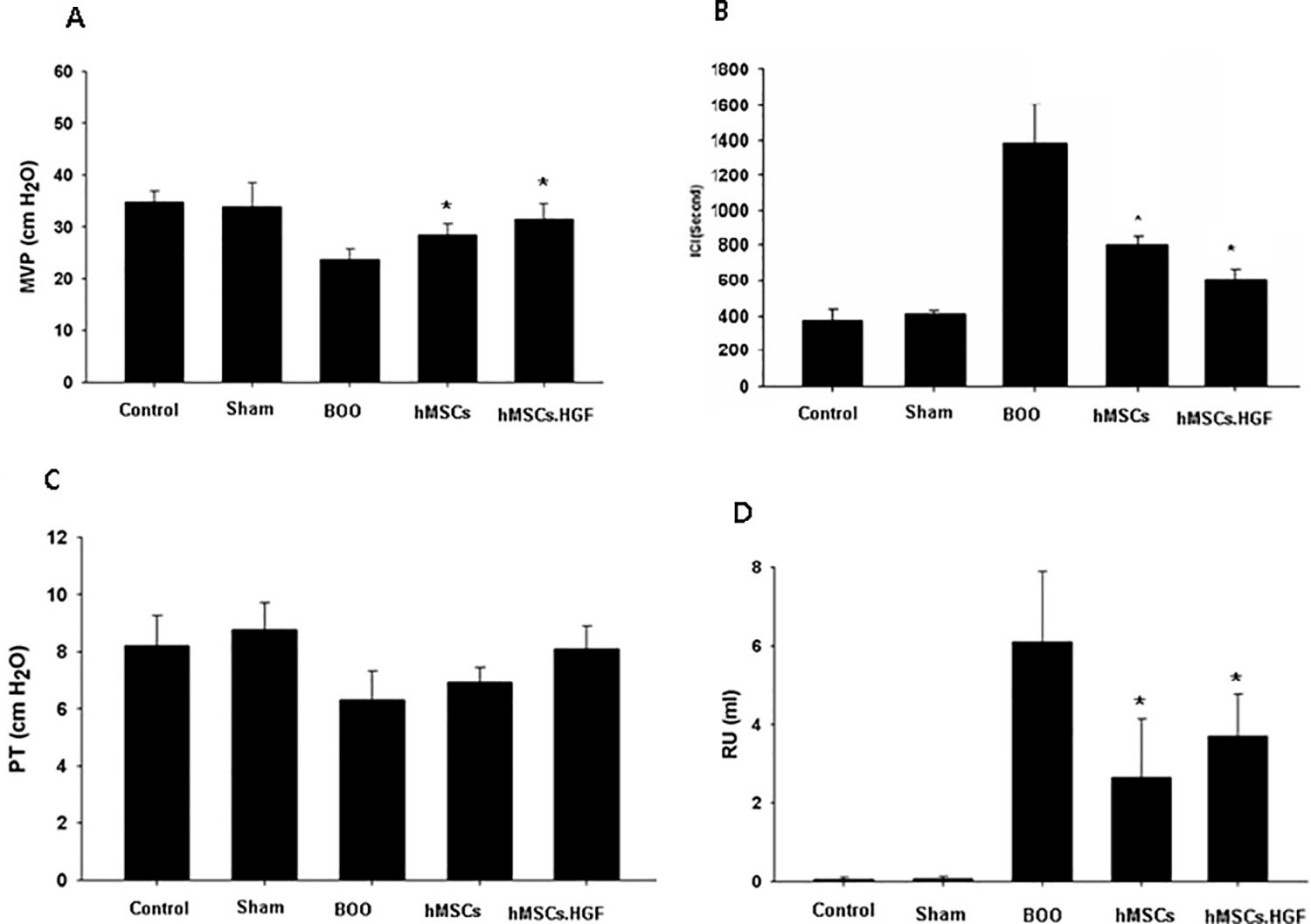
Recovery of cystometric parameters after transplantation. A. ICI increased after BOO and recovered after transplantation of hMSCs. B. MVP decreased after BOO and recovered after transplantation of hMSCs. C. PT was not altered among groups. D. RU increased after BOO and recovered after transplantation of hMSCs. Sham = sham operation, BOO = bladder outlet obstruction, hMSCs = human mesenchymal stem cells, hMSCs.HGF = HGF overexpressing human mesenchymal stem cells, ICI = intercontraction interval, MVP = maximal voiding pressure, PT = pressure threshold (PT), RU = residual urine volume. * *p* < 0.05.

A post-BOO increase in inter-contraction interval was restored to normal levels following, transplantation of hMSCs and further decreased after transplanting HGF-overexpressing hMSCs (*p* < 0.05). No alteration in pressure threshold was observed between groups. A post-BOO reduction in maximal voiding pressure was restored normal after transplantation of hMSCs. Post-void residual urine volume was elevated after BOO. However post-void residual urine volume decreased (*p* < 0.05) to control levels after transplantation of hMSCs. Post-void residual urine volume was the lowest in the group transplanted with HGF-overexpressing hMSCs (*p* < 0.05) (Table 2, Fig 3).

**Table 2:** Recovery of bladder contractility after transplantation.

Variable	Control	Sham	BOO	hMSCs	hMSCs. HGF
**ICI (seconds)**	379±57	412±17	1386±221	745±75*	615±84*
**MVP (cmH2O)**	34.8±2.1	33.9±4.7	20.6±3.5	28.5±2.1*	30.5±3.6*
**PT (cmH2O)**	8.5±0.8	9.2±1.2	6.7±0.8	7.0±0.6	8.1±0.9
**RU (mL)**	0.05± 0.07	0.07± 0.06	6.1±1.8	3.7±1.5*	3.0±1.2*

Sham = sham operation, BOO = removal of bladder outlet obstruction at 8 weeks after bladder outlet obstruction, hMSCs = human mesenchymal stem cells injected into the bladder wall, hMSC.HGFs = HGF overecpressing human mesenchymal stem cells injected into the bladder wall, ICI = inter-contraction interval, MVP = maximal voiding pressure, PT = pressure threshold, RU = residual urine volume.

*p < 0.05 value = BOO vs. BOO + hMSCs, BOO vs. BOO + hMSCs overexpressing HGF.

### mRNA expression in the bladder of BOO group

**Fig 4:**
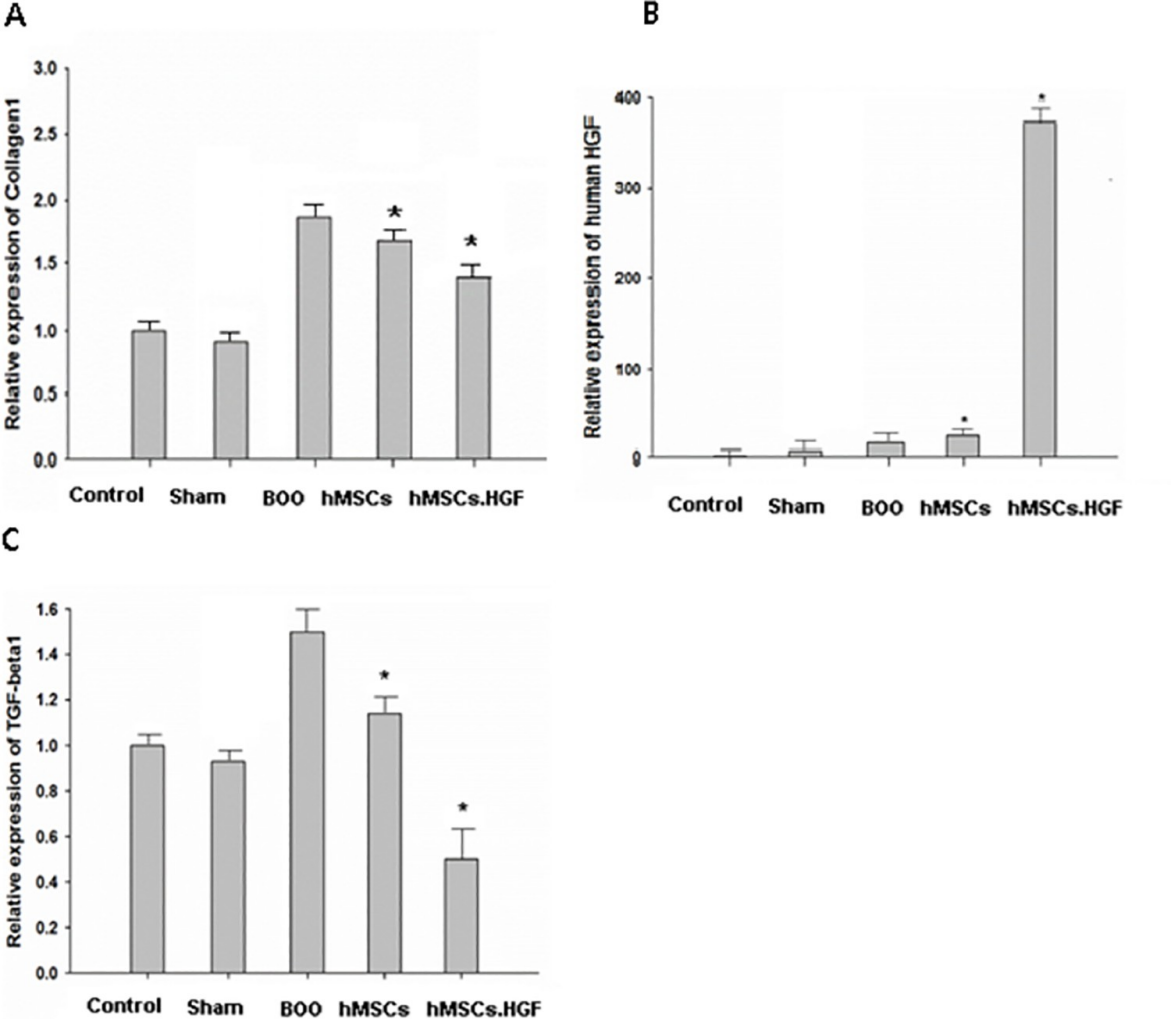
Expression of collagen1 and HGF mRNA. A. The mRNA expression of collagen1 increased in the group with BOO, which was recovered after transplantation of hMSCs.HGF. B. The mRNA expression of HGF increased in the group transplanted with hMSCs.HGF. C. The mRNA expression of TGF-β1 increased in the group with BOO, which was restored after transplantation of hMSCs.HGF. Sham = sham operation, BOO = bladder outlet obstruction, hMSCs = human mesenchymal stem cells, hMSCs.HGF = HGF overexpressing human mesenchymal stem cells injection. * *p* < 0.05.

The level of collagen 1 mRNA expression was increased in the BOO group and decreased after transplantation of hMSCs. It was further decreased (*p* < 0.05) after transplanting HGF-overexpressing hMSCs. HGF mRNA expression was increased (*p* < 0.05) after transplanting HGF-overexpressing hMSCs. The expression of TGF-β1 mRNA was increased in the BOO group and decreased after transplanting hMSCs. It was further decreased (*p* < 0.05) after transplanting HGF-overexpressing hMSCs (Fig 4).

### Histopathology

**Fig 5:**
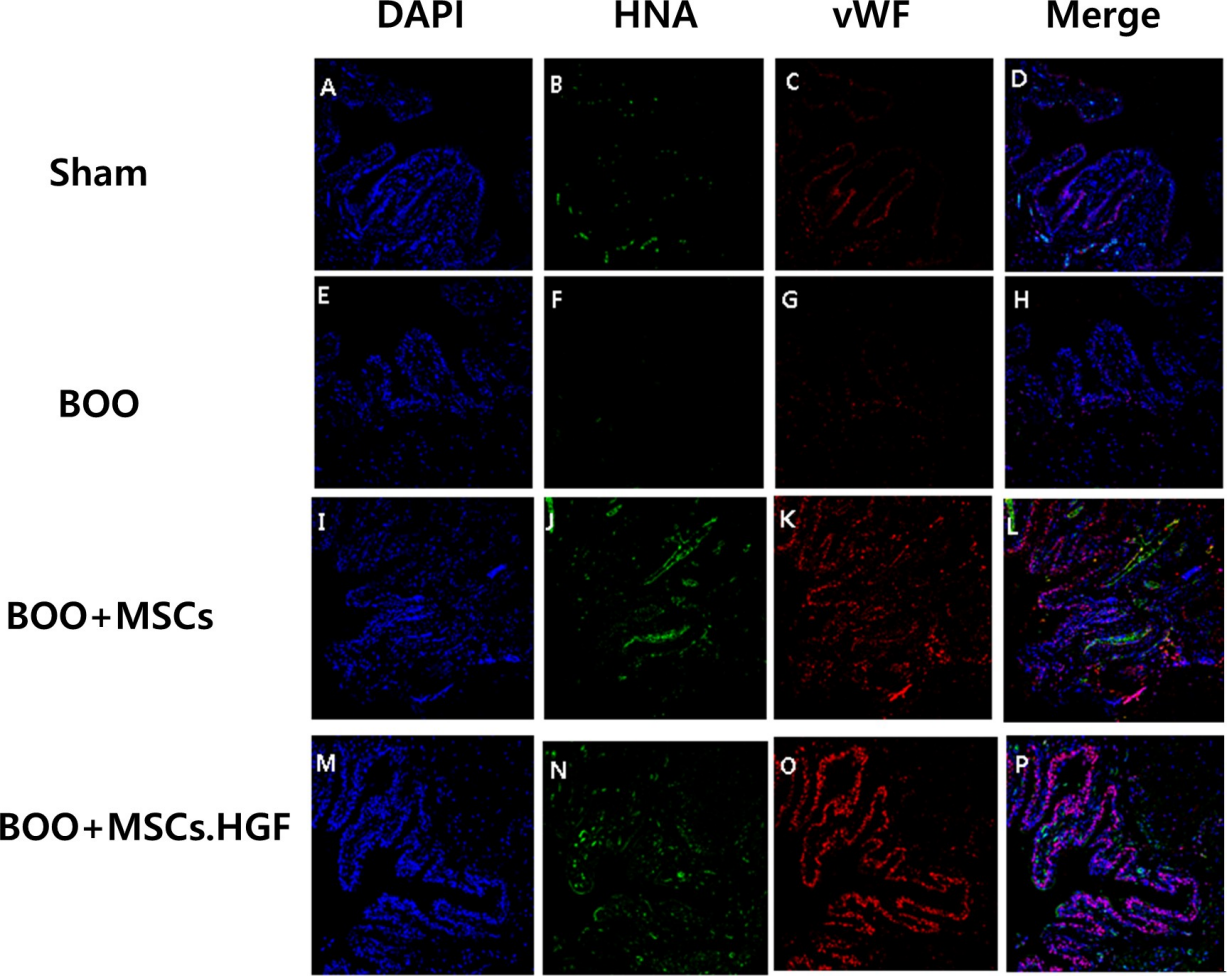
Immunofluorescent staining with vWF. Immunofluorescent staining with vWF showed a decrease in the number of vWF-positive cells in the group with BOO and an increase in vWF-positive cells after transplantation of hMSCs. The effect was enhanced by hMSCs.HGF. The merged cells expressing yellow fluorescence represent HNA and vWF-positive cells derived from hMSCs. A-D. Sham operation group. E-H. BOO group. I-L. hMSCs transplantation after BOOp. M-P. hMSCs.HGF transplantation after BOO. DAPI = 4’,6-diamidino-2-phenylindole, HNA = human nuclear antigen, vWF = von Willebrandt factor. Sham = sham operation, BOO = bladder outlet obstruction, hMSCs = human mesenchymal stem cells, hMSCs.HGF = HGF overexpressing human mesenchymal stem cells.

Immunofluorescent staining with vWF showed a decrease in the percentage of vWF-positive cells in the group of manifesting BOO. It was increased after transplantation of hMSCs and further increased after transplanting HGF-overexpressing hMSCs. Merged cells showing yellow fluorescence tested positive for human nuclear antigen (HNA) and vWF derived from hMSCs, indicating that hMSCs differentiated into endothelial cells (Fig 5).

**Fig 6:**
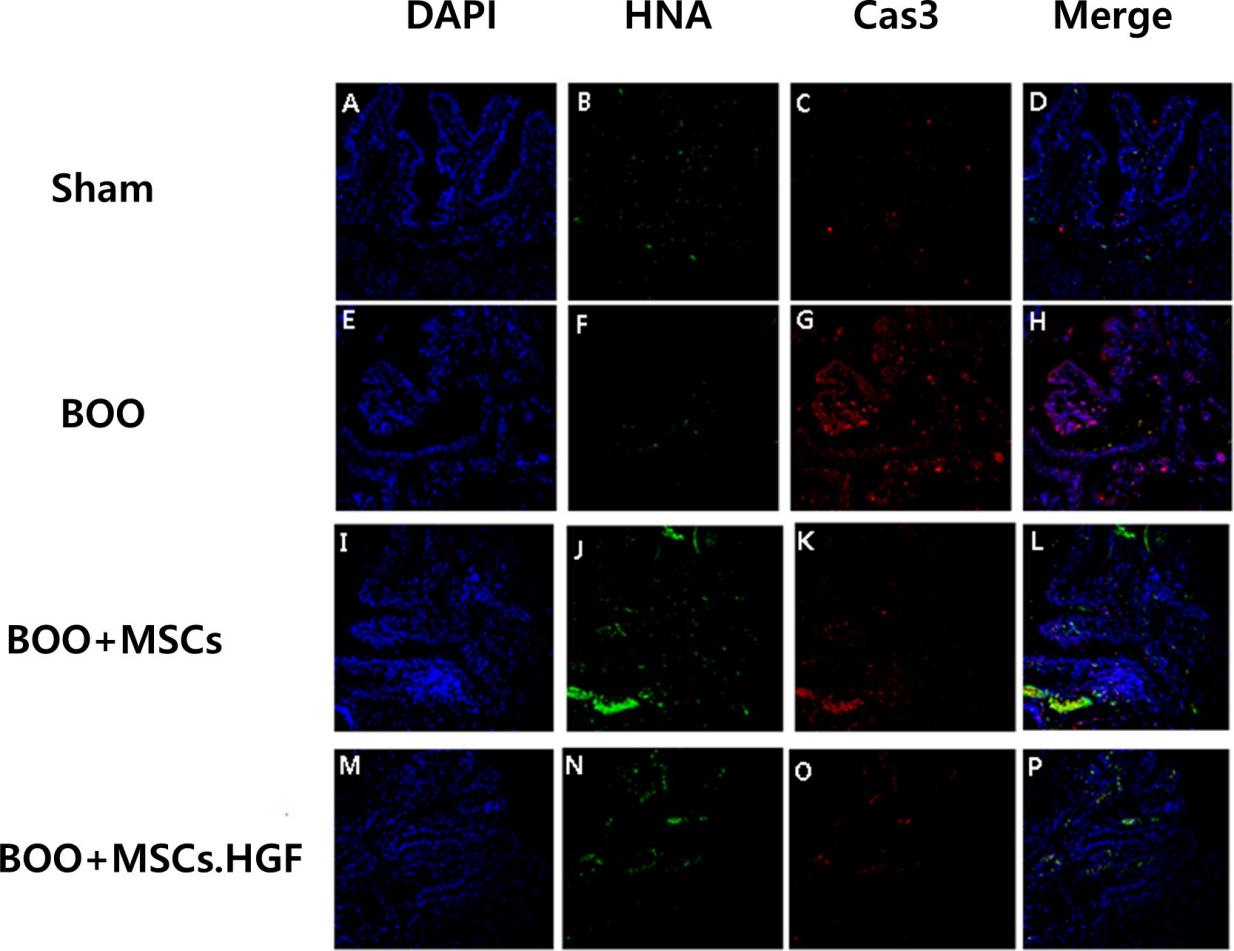
Immunofluorescent staining of bladder tissues. Staining with Cas3 revealed increased level of Cas3-positive apoptosis in the group with BOO and decreased Cas3-positive apoptosis following transplantation of hMSCs. The effect was enhanced by hMSCs.HGF. A-D. Sham operation group. E-H. BOO group. I-L. transplantation of hMSCs after BOO. M-P. hMSCs.HGF transplantation after BOO. DAPI = 4’,6-diamidino-2-phenylindole, HNA = human nuclear antigen, Cas3 = caspase 3. Sham = sham operation, BOO = bladder outlet obstruction, hMSCs = human mesenchymal stem cells, hMSCs.HGF = HGF overexpressing human mesenchymal stem cells.

**Fig 7:**
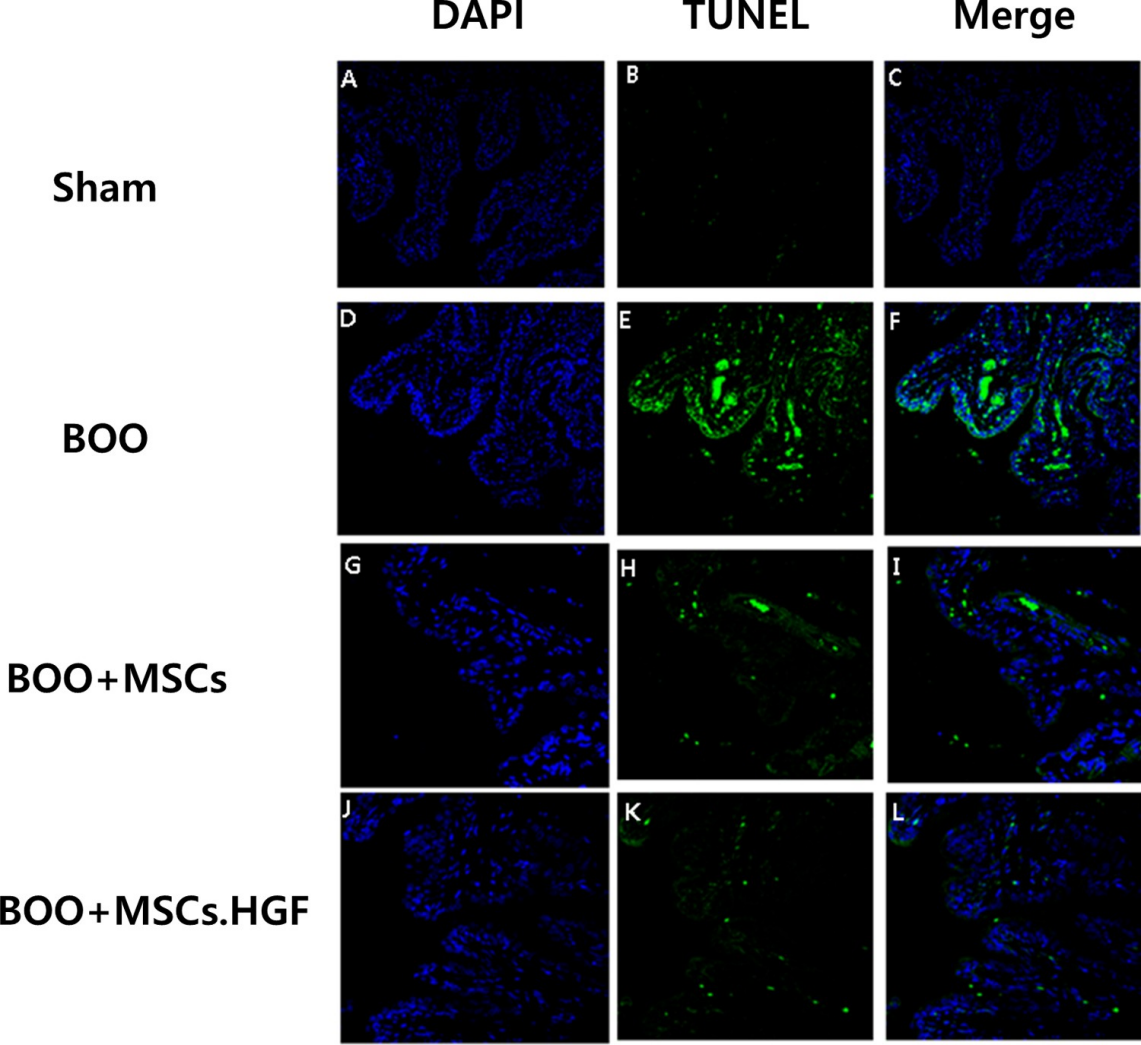
Immunofluorescent staining of bladder tissues. Staining with TUNEL led to increased TUNEL-positive apoptosis in the group of rats with BOO and decreased TUNEL-positive apoptosis after transplantation of hMSCs. The effect was enhanced by hMSCs.HGF. A-C. Sham operation group. D-F. BOO group. G-I. hMSCs transplantation after BOO. J-L. hMSCs.HGF transplantation after BOO. DAPI = 4’,6-diamidino-2-phenylindole, TUNEL = deoxynucleotidyl transferase-mediated dUTP nick end labeling, Sham = sham operation, BOO = bladder outlet obstruction, hMSCs = human mesenchymal stem cells, hMSCs.HGF = HGF overexpressing human mesenchymal stem cells.

Immunofluorescent staining revealed an increase in the levels of Cas3 and TUNEL-positive apoptosis in the group with BOO. Transplantation of hMSCs led to a decrease in the levels of Cas3 and TUNEL-positive apoptosis and further decrease after transplantation of HGF-overexpressing hMSCs (Figs 6 and 7).

## Discussion

In this study, prolonged BOO led to increased bladder weight, enhanced collagen deposition, prolonged inter-contraction interval and elevated residual urine volume with a decrease of bladder contractility (Figs 1–3), suggesting that successful induction of underactive bladder. We transplanted hMSCs or HGF-overexpressing hMSCs into underactive bladder walls of rats experiencing BOO to restore bladder contractility.

Transplantation of hMSCs decreased the interval between contractions, increased the amplitude of bladder contractions, and decreased the residual urine volume suggesting successful restoration of bladder contraction which was further improved after transplanting hMSCs overexpressing HGF (Fig 3).

Several different types of stem cells are used to treat bladder dysfunction in animal models. All MSCs, including BM-MSCs, adipose-derived stem cells (ADSCs) and skeletal muscle derived stem cells (Sk-MSCs) exhibit similar biological properties and capabilities; however, their availability depends on the therapeutic purpose. BM-MSCs and Sk-MSCs require prolonged expansion time with complex isolation procedures. In contrast, ADSCs are easily available with short expansion time [19]. In our previous study, we used BM-MSCs [17, 18]. A study by Zhang et al. [20] using ADSCs injected into the detrusor or via tail vein reported alleviation of voiding dysfunction compared with the control group in a rat model of DM-induced detrusor dysfunction. Nitta et al. [21] used Sk-MSCs in a bladder dysfunction model after pelvic plexus injury. Autologous stem cell transplantation around the damaged region significantly enhanced the functional recovery (78%) compared with the controls. In this study, we investigated that the effects could be enhanced by overexpressing HGF in hMSCs.

Bladder obstruction is primarily characterized by cellular proliferation and hypertrophy as well as extracellular matrix aggregation [1, 2]. Obstruction increases bladder wall thickness and weight [3, 4]. MSCs are known to reduce cardiac and liver fibrosis [11, 14]. Collagen deposition was increased in the bladder of the BOO rat model, but restored to normal level after transplanting hMSCs (Fig 2). In the present study, real-time PCR showed that the level of bladder collagen 1 and TGF-β mRNA were increased in the rat model of BOO, but decreased after transplantation of hMSCs. These results suggest that transplantation of hMSCs improves bladder contractility in rats with underactive bladder by inhibiting collagen deposition. Transplantation of hMSCs overexpressing HGF further improved bladder contractility (Fig 4).

Endogenous and exogenous HGFs are known to exhibit antifibrotic activities [22, 23]. HGF activates tyrosine kinase and inhibits the expression of TGFβ1 and corresponding type I receptor TGFβR-1 [24–26]. The hMSC-derived HGF inhibits collagen deposition, which is enhanced by the potent release of HGF from hMSCs overexpressing HGF.

In this study, the hMSCs were validated using anti-human nuclear (HNA) antibody in bladder sections transplanted with hMSCs. HNA-positive cells were found in bladder walls of rats transplanted with hMSCs (Figs 5 and 6).

Transplanted hMSCs differentiate into smooth muscle cells [27]. Cell replacement represents a possible therapeutic mechanism. In the present study, the percentage of vWF-positive cells decreased in the BOO group, but increased after transplantation of hMSCs. Merged cells expressing both HNA and vWF derived from hMSCs, indicated differentiation of hMSCs into endothelial cells (Fig 5). This result suggests that transplantation of hMSCs can improve bladder contractility in rats with underactive bladder via cell replacement.

Wall thickening triggers cyclical ischemia/reperfusion with each micturition cycle [5, 6]. A direct correlation exists between reduced detrusor blood flow induced by BOO and degree of decompensation [7]. Decreased number of vWF-positive cells in BOO bladder suggests a decreased number of microvessels supplying bladder wall and decreased detrusor blood flow. In the present study, the percentage of vWF-positive microvessels increased in the BOO bladder after transplanting hMSCs compared with the sham operation group (Fig 5).

In this study, the group transplanted with hMSCs exhibited a post-BOO elevation in HGF protein expression, which was further enhanced by transplanting HGF-overexpressing hMSCs (Fig 4). HGF secreted by hMSCs protects tissue from ischemic injuries such as BOO. The hMSCs at the ischemic lesion sites in BOO can enhance bladder function by not only generating substantial levels of HGF, but also protecting the bladder and inducing neo-angiogenesis. Damaged bladder sites are replenished with blood, oxygen, and nutrients owing to HGF-facilitated angiogenesis, which was enhanced by overexpressing HGF in hMSCs (Fig 5).

BOO triggers ER stress via bladder hypoxia which may contribute to the activation of BOO-related apoptosis [8, 9]. In the present study, the expression of proapoptotic protein Cas3 was decreased in the ischemic bladder of BOO exposed to hMSCs and increased in the absence of exposure to hMSCs (Fig 6). In addition, the number of TUNEL-positive cells in muscles undergoing apoptotic cell death was decreased in rats with BOO treated with hMSCs than in untreated rats. It was further decreased after transplanting hMSCs overexpressing HGF (Fig 7). These results suggest that hMSCs can protect the bladder by down-regulating the expression of pro-apoptotic proteins. HGF exhibits protective activities mediated via anti-apoptotic signals [12, 13]. The effect of hMSCs was enhanced by overexpressing HGF (Fig 7). Under normal conditions, the HGF signaling pathway is generally silent. However, it is activated in organ injury as a protective mechanism against apoptosis, possibly facilitating wound healing and organ regeneration [13, 28, 29]. Both human and murine models of infarction showed elevated HGF levels in the cytoplasm [30, 31]. Furthermore, both endogenous and exogenous HGFs inhibit apoptosis [24, 25]. Thus, smooth muscle cell apoptosis may be prevented by hMSC-derived HGF. In this study, a post-BOO rise in TGFβ protein expression was restored to normal following transplantation with hMSCs. Taken together, these results demonstrate that transplanting hMSCs provides bladder protection in rats experiencing BOO by downregulating pro-apoptotic protein expression. Such protective effect can be enhanced by overexpressing HGF in hMSCs.

## Conclusions

Transplantation of hMSCs into bladder walls increases neo-angiogenesis, decreases collagen deposition and apoptosis of detrusor muscles, and improves bladder underactivity. Such effects are enhanced by overexpressing HGF in hMSCs. These results suggest that recovery of bladder underactivity using hMSCs may also be useful in ameliorating micturition disorders in patients with underactive bladder due to prolonged BOO. Further studies are needed before clinical applications can be envisaged.

## Supporting information

S1 File (ZIP)
