# Correction: Improved bladder contractility after transplantation of human mesenchymal stem cells overexpressing hepatocyte growth factor into underactive bladder from bladder outlet obstruction models of rats

**DOI:** 10.1371/journal.pone.0269577

**Published:** 2022-06-02

**Authors:** The PLOS ONE Staff

In the Funding section, the grant number from the funder Ministry of Education, Science and Technology is listed incorrectly. The correct grant number is: 2021R1A2C1004163. The publisher apologizes for the error.
